# Quantitative MRI phenotypes capture biological heterogeneity in multiple sclerosis patients

**DOI:** 10.1038/s41598-021-81035-8

**Published:** 2021-01-15

**Authors:** Ide Smets, An Goris, Marijne Vandebergh, Jelle Demeestere, Stefan Sunaert, Patrick Dupont, Bénédicte Dubois

**Affiliations:** 1 Laboratory for Neuroimmunology, Department of Neurosciences, KU Leuven, Herestraat 49, Box 1022, 3000 Leuven, Belgium; 2 Leuven Brain Institute KU Leuven, Leuven, Belgium; 3 Department of Neurology, University Hospitals Leuven, 3000 Leuven, Belgium; 4 Department of Imaging and Pathology, Translational MRI, KU Leuven, 3000 Leuven, Belgium; 5 Department of Neurosciences, Laboratory for Cognitive Neurology, KU Leuven, 3000 Leuven, Belgium

**Keywords:** Neurological disorders, Multiple sclerosis

## Abstract

Magnetization transfer ratio (MTR) and brain volumetric imaging are (semi-)quantitative MRI markers capturing demyelination, axonal degeneration and/or inflammation. However, factors shaping variation in these traits are largely unknown. In this study, we collected a longitudinal cohort of 33 multiple sclerosis (MS) patients and extended it cross-sectionally to 213. We measured MTR in lesions, normal-appearing white matter (NAWM), normal-appearing grey matter (NAGM) and total brain, grey matter, white matter and lesion volume. We also calculated the polygenic MS risk score. Longitudinally, inter-patient differences at inclusion and intra-patient changes during follow-up together explained > 70% of variance in MRI, with inter-patient differences at inclusion being the predominant source of variance. Cross-sectionally, we observed a moderate correlation of MTR between NAGM and NAWM and, less pronounced, with lesions. Age and gender explained about 30% of variance in total brain and grey matter volume. However, they contributed less than 10% to variance in MTR measures. There were no significant associations between MRI traits and the genetic risk score. In conclusion, (semi-)quantitative MRI traits change with ongoing disease activity but this change is modest in comparison to pre-existing inter-patient differences. These traits reflect individual variation in biological processes, which appear different from those involved in genetic MS susceptibility.

## Introduction

Multiple sclerosis (MS) is the most common disease of the central nervous system (CNS) in young adults, and may lead to serious physical and cognitive disability. The disease course is distinguished as presenting with relapsing–remitting disease at onset (bout onset MS), which may over time convert to a progressive disease, or with progression from onset (primary progressive MS). Important patient-to-patient heterogeneity is seen for clinical (e.g. onset, severity) as well as paraclinical (e.g. laboratory measures, imaging) features. MS affects white and grey matter in the CNS through inflammation, demyelination and axonal degeneration^1^. Several conventional and non-conventional imaging outcomes reflect these hallmarks^2^. T2 MRI sequences with or without gadolinium administration are typically used to assess inflammatory brain lesions and their evolution. Volumetric brain measurements quantify gross tissue loss and represent an MRI biomarker of neurodegeneration^3^. Finally, a decreased magnetization transfer ratio (MTR), both in lesions and in normal-appearing white matter (NAWM) or grey matter (NAGM), correlates with reduced myelin content post-mortem^4^ and with remyelination^5^, although additional pathologic features may contribute to the MTR signal.

Both brain volume loss and subtle reductions in MTR are apparent early in MS^6^. Nevertheless, there are striking patient-to-patient differences. Some patients withstand neurodegeneration better due to differences in brain and cognitive reserve^7,8^, and remyelination can be considerable in some cases while virtually absent in others^9^. Most currently used immunomodulating drugs at best only modestly influence the rate of brain volume loss. To date, we do not understand why patients exhibit these different levels of MTR reduction and brain volume loss. Moreover, the influence of MS susceptibility genes on these MRI phenotypes is largely unknown. Therefore, it is unclear whether the immunological pathways influencing susceptibility also shape (semi-)quantitative MRI traits^10^. Such understanding would have important implications for neuroprotective and remyelinating treatment strategies.

Hence, we studied in a longitudinal cohort whether variation in MTR and volumetric indices over time is (1) primarily influenced by inter-patient demographic differences or by the ongoing MS disease activity. In a cross-sectional cohort, we scrutinized (2) whether there is a correlation between MTR values in white, grey matter and lesions and to which extent these MTR values are influenced by (3) demographic variables and (4) the MS genetic risk score.

## Materials and methods

### Study population

We included a cross-sectional study population of 213 Caucasian patients, 209 of whom were finally eligible for genetic analysis (cfr. infra). Longitudinal MRI data obtained on the same and most frequently used scanner protocol were available for 33 of 213 patients. Patients were diagnosed with MS according to the McDonald 2010 criteria^11^ in University Hospitals Leuven, underwent imaging between July 2012 and February 2017, and donated a blood sample for genetic analysis. Patients provided written informed consent, and the Ethics committee of the University Hospitals Leuven approved the study (S60222). All research was performed in accordance with their guidelines. Clinical data were collected during follow-up by the same expert treating clinician (B.D.). Multiple Sclerosis Severity Score (MSSS) was calculated based on the Expanded Disability Status Scale (EDSS) and disease duration^12^.

### MRI and image analysis

We obtained and analysed the images as we described previously^13^. MRI data were acquired on a 3T MRI scanner (Intera, Ingenia, or Achieva; Philips, Best, The Netherlands) equipped with an 8-, 15- or 32-channel head coil using parameters corresponding to 6 different MRI protocols as summarized in Supplementary Table S1. All sequences were obtained in the context of routine clinical follow-up at the Neurology Department of University Hospitals Leuven. The magnetization transfer (MT) imaging data were obtained by acquiring 2 axial gradient-echo images with and without an off-resonance magnetization transfer saturation pulse. From the MT sequences, we calculated the MTR value as MTR = 100 × (M_0_ − M_s_)/M_0_, where M_s_ and M_0_ represent the signal intensity with and without application of the saturation pulse, respectively. The MTR and 3-dimensional (3D) fast fluid-attenuated inversion recovery (FLAIR) images were co-registered to the 3D-T1 weighted images using statistical parametric mapping (SPM; Welcome Trust Centre for Neuroimaging, version SPM12). Next, we applied the lesion segmentation toolbox using 20 thresholds for kappa ranging from 0 to 1 in steps of 0.05^14^. This parameter controls the initialization of a lesion-growing algorithm. Low values of kappa lead to segmentations of lesions, which can be considered more sensitive but less specific for real lesions, and the reverse is true for high values of kappa. We used a kappa of 1 for lesion segmentation. This lesion segmentation toolbox requires the 3D T1 and FLAIR images as input and provides the lesion segmentation as well as a hard segmentation in grey matter, white matter and cerebrospinal fluid (CSF) based on the VBM8 toolbox (University of Jena). Using this hard segmentation, we defined NAGM and NAWM as voxels belonging to grey and white matter, respectively, but not belonging to lesions when kappa = 0. We calculated MTR values of the co-registered image for each tissue class (NAGM, NAWM, and lesions), plotted them in a histogram and extracted histogram parameters such as median, peak height (expressed as unit percent), peak location and the mean of the middle 90 percentiles (mean90). We filled the lesions in the 3D-T1 image and soft segmented this image using SPM12 with the default settings. We calculated volumes of grey and white matter and CSF as the sum of the soft segmentation classes multiplied with voxel volume. We defined total brain volume as the sum of the volumes of these three compartments. White matter and grey matter volume were expressed as a percentage of total brain volume.

### Imaging-related statistical analysis

For the cross-sectional study population, we used the first available scan of each patient. For the longitudinal cohort, we considered only patients scanned at least twice using the same and most frequently used scanner protocol. Due to the high inter-correlation between histogram parameters (Supplementary Table S2), we included only median and peak height MTR in the statistical analysis with Rv3.6.1^15^. For the cross-sectional cohort, we analysed 10 MRI parameters (median and peak height MTR in lesions, NAWM and NAGM as well as WM, GM, total brain and lesion volume) in function of demographic (gender, age), clinical (disease duration, OCB status, IgG index, MSSS and treatment status), and genetic (HLA, non-HLA and total genetic burden) parameters. MRI protocol is always added as categorical covariate, and age and gender as a continuous covariate for all clinical and genetic analyses. We calculated the percentage of variance in MRI traits explained by age and gender by subtracting the adjusted r^2^ from the full model (MRI ~ Age + Gender + MRI Protocol) with the adjusted r^2^ of the baseline model (MRI ~ MRI Protocol).

To quantify intra- versus inter-patient variation in imaging parameters as done previously for immunological parameters^16^, we regressed the MRI parameters in function of two generalized linear models. A generic generalized linear model (MRI ~ Patient identifier code + Time between interval scans) specifically includes a patient identifier such that it will generate a different intercept for each patient. We calculated the contribution of the inter- (patient identifier) and intraindividual (time) variation to explained variance in MRI traits in the generic model with the relaimpo package by subtracting the adjusted r^2^ of the baseline generic model without one variable from the adjusted r^2^ of the full generic model^16,17^. After establishing that intra-individual variation is low compared to inter-individual variation, we subsequently analyzed whether demographical and clinical parameters age, gender and disease duration can (partially) replace or account for patient identifier as fixed effects in a clinical generalized linear model (MRI ~ Age + Gender + Disease duration + Time between interval scans) or whether other, unknown parameters should be invoked to explain inter-individual variation.

Bonferroni multiple testing correction starting from an overall targeted type I error rate of 5% was applied separately for the longitudinal cohort (P ≤ 0.0025 for the 10 MRI measures and 2 models described above) and cross-sectional study population (P ≤ 0.00047 for 10 MRI measures vs. 10 demographic/clinical/genetic variables described above and 6 tests of median and peak height MTR across the three tissue classes).

### Genotyping, quality control and imputation

DNA was extracted from total blood using standard methods with an in-house protocol. Genotyping for 700,078 variants using the Infinium HTS assay on Global Screening Array bead-chips (Illumina) followed by genotype calling and quality control (QC) using PLINK v1.9 was done as described previously^13,18,19^. For one patient no genetic data were available, and three samples were excluded because of cryptic relatedness (identity by descent > 0.1875). This left a total of 209 patients remaining in the genetic analysis. In the cleaned sample set we performed variant QC and excluded variants with minor allele frequency < 1%, call rate < 98% and significant deviation from Hardy–Weinberg equilibrium (P < 10^–6^). A total of 502,527 SNPs remained in the analysis. Strand alignment, pre-phasing and imputation were done as described previously^13,18^. Classical Human Leukocyte Antigen (HLA) alleles, amino acid polymorphisms and SNPs were imputed with SNP2HLA v1.0.3 and the T1DGC reference panel (build 37)^20^.

### Polygenic risk score

The latest MS genomic map includes 138 primary, independent autosomal MS risk factors outside the HLA region as well as 31 statistically independent associations within the HLA region^10^. The primary autosomal variants were identified in the marginal analysis, i.e. no other variant was included in the model (step 0). The secondary, conditional variants (not included in our MS genetic risk score), were identified in a step-wise modelling approach, including variants from previous steps^10^. Overall, 133 primary, independent autosomal SNPs (including 5 proxy SNPs with r^2^ > 0.9) as well as 22 statistically independent risk variants in the HLA region could be extracted from the imputed genetic data (Supplementary Table S4–S5). All SNPs had a MAF > 1% and genotype imputation info score ≥ 0.8 or genotype imputation r^2^ ≥ 0.8 for respectively non-HLA or HLA SNPs. Subsequently, we calculated the polygenic risk score for each patient with PRSice v2.3.1.e by summing the allele dosage for each variant weighted by the logarithm of the odds ratio obtained for that variant in the original susceptibility study^10^. For each genetic variant, alleles were aligned and matched so that their effects correspond to an increase in MS risk. Linear regression analyses for polygenic risk scores with MRI parameters were performed in Rv3.6.1, including age at MRI, sex and MRI protocol as covariates. Single SNP frequentist association tests for N = 133 non-HLA and N = 22 HLA variants with 10 MRI parameters as variables and age at MTR, sex and MRI protocol as covariates were performed with SNPTEST v2.5.2^21^.

### Data availability

The raw data are available at KU Leuven and will be shared upon request from any qualified investigator pending Institutional Review Board approval and accordance with EU General Data Protection Regulation.

## Results

### High inter-patient differences with low variation over time characterize MRI traits

**Figure 1:**
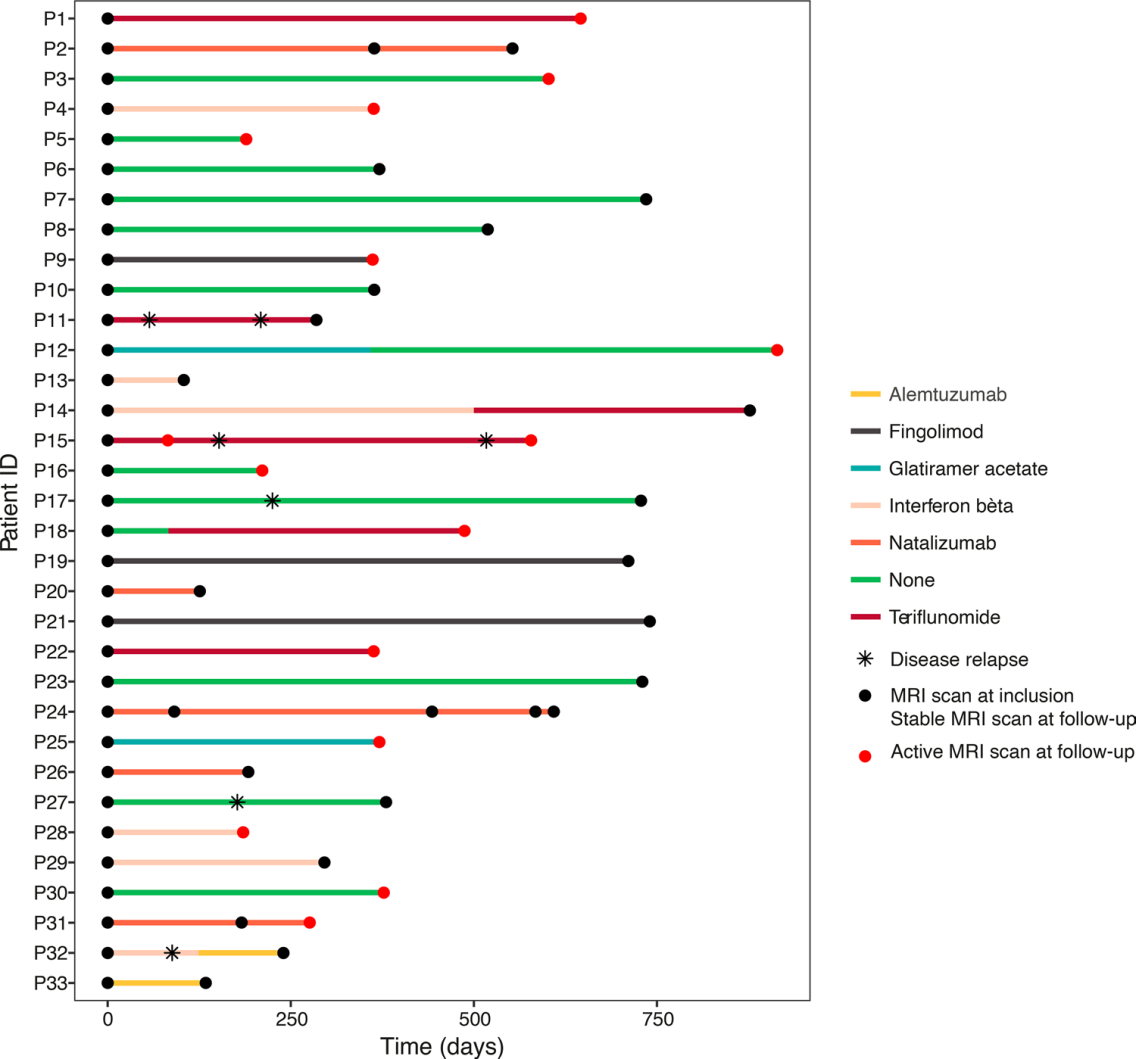
Timeline of treatment modality, clinical relapses and MRI scans of the longitudinal cohort. Patients included in the longitudinal cohort (N = 33) were scanned at least twice using the same MRI protocol D (72 scans in total: N = 29 had two scans, N = 3 had three scans, and N = 1 had five scans). The radiology reports that were generated in the context of routine clinical follow-up at the University Hospitals Leuven allowed to distinguish between an ‘active’ and ‘stable’ MRI scan at follow-up. Whenever the neuroradiologists mentioned gadolinium enhancement, new lesions or lesions with an increased volume compared to the preceding MRI scan this implied coding of the MRI scan as ‘active’ (Image generated with Rv3.6.1, https://www.R-project.org).

**Figure 2:**
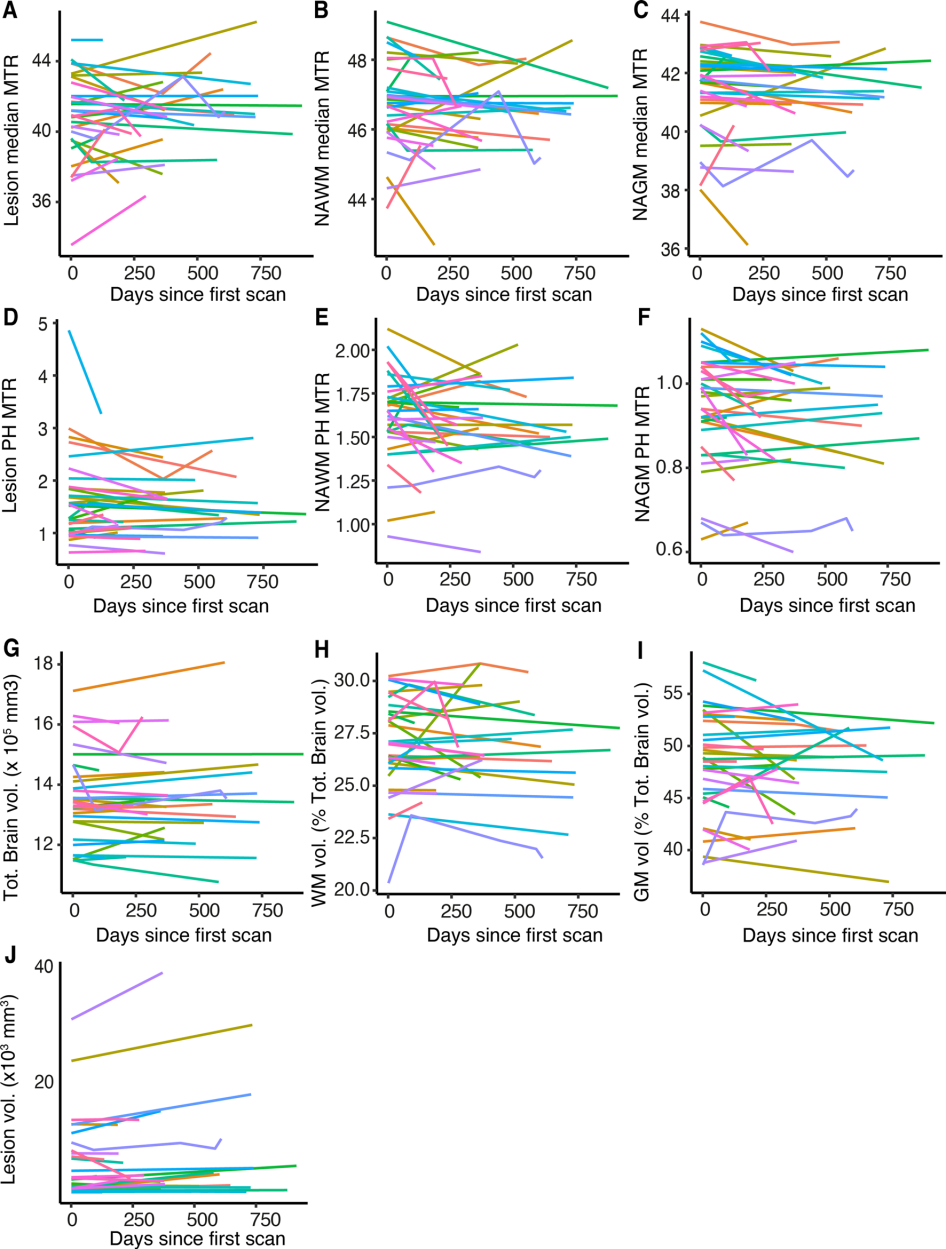
Longitudinal variation in MRI. Longitudinal evolution of (**A**–**C**) median and (**D**–**F**) peak height MTR in each tissue class (lesions, NAWM, NAGM) as well as (**G**) total brain volume, (**H**) white matter, (**J**) grey matter and (**I**) lesion volume. The same color represents each individual patient (N = 33) throughout the graphs. All patients were scanned using the same scanner protocol. *MTR *magnetization transfer ratio, *NAWM *normal appearing white matter, *NAGM *normal appearing grey matter, *PH *peak height, *Tot. *total, *vol. *volume, *WM *white matter, *GM *grey matter (Image generated with Rv3.6.1, https://www.R-project.org).

**Figure 3:**
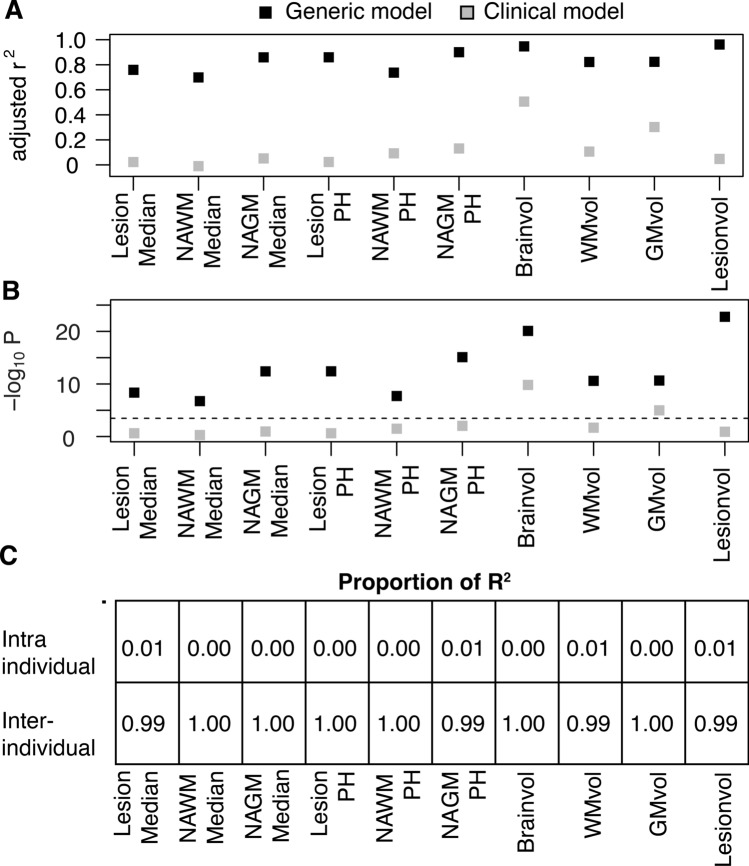
Contribution of inter- and intra-individual variance in longitudinal MRI data. In the longitudinal cohort of 33 patients we visualized the (**A**) percentage of variance explained (= adjusted r^2^) and (**B**) significance in a generic model (black) including a unique patient identifier code (i.e. inter-individual variation) and time (i.e. intra-individual variation) and a clinical model (grey) including gender, age, disease duration (i.e. known variables of inter-individual variation) and time. The dotted line represents the cut-off for significance of P ≤ 0.05 after correction for multiple testing (P ≤ 0.0025). (**C**) The relative contributions of inter-individual and intra-individual variation over time to the total variance explained in the generic model. *PH *peak height, *NAWM *normal appearing white matter, *NAGM *normal appearing grey matter, *WM *white matter, *GM *grey matter, *vol *volume (Image generated with Rv3.6.1, https://www.R-project.org).

For the longitudinal cohort of 33 patients, we obtained two to five scans with the same scanner protocol and with an average interval of 13.2 ± 7.9 months between the first and second scan (Table 1). Figure 1 depicts the timing of MRI scans, clinical relapses during follow-up as well as the applied treatment strategy. In the longitudinal cohort, we visualized the evolution over time of all included MRI traits in Fig. 2. Next, we modelled the longitudinally obtained imaging parameters in function of a unique patient identifier code (reflecting inter-patient demographic variation) and the number of days since the first scan (reflecting intra-patient variation in disease activity over time). This generic model explained > 70% of variance in MTR (P ≤ 1.85 × 10^–7^) and > 82% of variance in volumetric measures (P ≤ 2.57 × 10^–11^) (Fig. 3A,B). Inter-patient differences at inclusion (≥ 99%) determine the majority of the explained variance whereas intra-patient heterogeneity contributed only modestly (≤ 1.0%) (Fig. 3C).

**Table 1:** Demographics of the study population.

Demographical/clinical characteristics	Cross-sectional	Longitudinal
Number of patients included	213	33
Gender: N female/male (% female)	151/62 (70.9%)	24/9 (72.7%)
Age at onset (years): median (range)	29 (4–68)	27 (4–68)
Disease course: Bout onset/primary progressive/unknown (% bout onset)	208/2/3 (97.6%)	33/0/0 (100.0%)
Disease duration (years): median (range)	8 (0–43)	6 (1–20)
Age at imaging (years): median (range)	39 (19 -71)	36 (19–69)
Interval between first and second scan (months): mean (standard deviation)	–	13.2 (7.9)
MSSS at imaging: median (range)	1.61 (0.11–9.45)	1.21 (0.19–8.50)
Oligoclonal bands: positive/negative/unknown (% positive)	175/18/20 (90.6%)	26/4/3 (86.7%)
IgG index: median (range)	0.90 (0.44–4.2)	0.94 (0.5–4.2)
**Therapy at imaging: N (%)**	133 (62.5%)	21 (63.6%)
None	80 (37.5%)	12 (36.4%)
Interferon-beta	62 (29.1%)	6 (18.1%)
Glatiramer acetate	21 (9.9%)	2 (6.1%)
Teriflunomide	15 (7.0%)	4 (12.1%)
Fingolimod	13 (6.1%)	3 (9.1%)
Natalizumab	14 (6.6%)	5 (15.2%)
Other (dimethyl fumarate and alemtuzumab)	8 (3.8%)	1 (3.0%)

Subsequently, we investigated whether known demographical and clinical patient-specific parameters explained inter-patient variation. For this purpose, we analyzed the longitudinal imaging parameters in function of known variables of inter-patient variation (gender, age, disease duration at inclusion) and the number of days since the first scan. The three patient-specific covariates clarified substantially less of variance in the total brain and grey matter percentage (r^2^ ≤ 0.51) compared to the generic model (r^2^ ≥ 0.87). Moreover, a model based on known patient-specific factors could not significantly explain variance in lesion volume, white matter percentage and MTR parameters (r^2^ ≤ 0.10, P ≥ 0.007) (Fig. 3A,B).

### MTR traits correlate across tissues within individual patients

**Figure 4:**
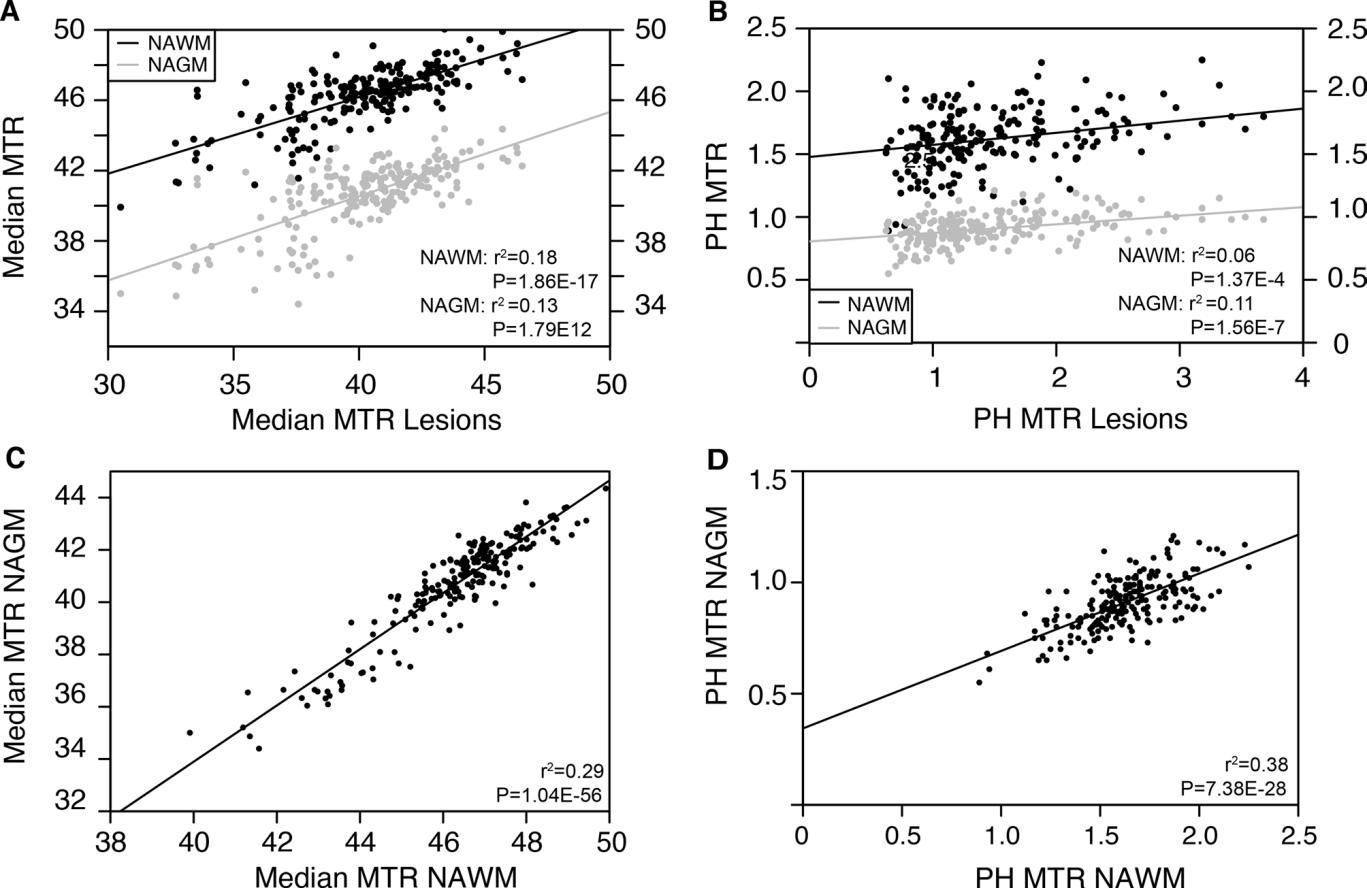
Correlation of MTR over tissue classes within patients. Correlation of (**A**) median and (**B**) peak height MTR in normal appearing tissues versus median and peak height MTR in lesions. Correlation between (**C**) median and (**D**) peak height MTR in NAWM versus NAGM in the cross-sectional study population (N = 213). Adjusted r^2^ and P-values result from linear regression with covariates age, gender and MRI protocol. The Bonferroni cut-off for significance of P ≤ 0.05 after correction for multiple testing was 0.00047. *MTR *magnetization transfer ratio, *NAWM *normal appearing white matter, *NAGM *normal appearing grey matter, *PH *peak height (Image generated with Rv3.6.1, https://www.R-project.org).

In order to increase power to investigate relevant correlations, we extended our cohort to 213 MS patients with available cross-sectional MRI data (Table 1). The majority of these patients were female (70.9%) and had a relapsing–remitting disease course (97.6%). The median age at imaging was 39 years with a median disease duration of 8 years. In the cross-sectional population, we noted a highly significant correlation between MTR values of different tissue classes (Fig. 4). Most prominently, MTR correlated moderately between the two types of normal-appearing tissue, NAWM and NAGM (median: r^2^ = 0.29, P = 1.04 × 10^–56^; peak height: r^2^ = 0.38, P = 7.38 × 10^–28^). MTR reductions in lesions paralleled to a lesser extent MTR decrease in NAWM and NAGM (median: r^2^ = 0.18–0.13, P ≤ 1.79 × 10^–12^; peak height: r^2^ = 0.06–0.11, P ≤ 1.37 × 10^–4^). These findings highlight correlation in MTR traits across tissues.

### Demographic and clinical variables modestly shape MRI traits

Median and peak height MTR showed substantial variability among patients in lesions, NAWM and NAGM (Fig. 4). Female gender correlated highly significantly with lower brain volume (P = 1.67 × 10^–21^) and higher grey matter percentage (P = 3.58 × 10^–4^) whereas increasing age correlated with lower grey matter percentage (P = 4.57 × 10^–16^) (Supplementary Table S3). Age and gender together determined 34 percent of the variance in total brain volume and 30 percent of variance in grey matter percentage. These observations reflect known physiological correlations and provide validation for our study. Increasing age was associated with a decrease in peak height MTR, surviving multiple testing for NAGM and with a trend for NAWM and lesions. Age and gender together determined between 0.6 and 2.0% of the total variance in median MTR traits and 4.4 and 8.4% of total variance in peak height MTR traits, respectively. The MSSS correlated significantly with increased lesion volume (P = 1.19 × 10^–4^). No associations remained significant after correction for multiple testing with regard to disease duration (P ≥ 0.02), presence of oligoclonal bands (P ≥ 0.07), IgG index (P ≥ 0.13) and treatment status (P ≥ 4.87 × 10^–4^). Altogether, demographic and clinical variables only account for at most 10% or one third of inter-patient heterogeneity in MTR and volumetric traits, respectively.

### The MS genetic risk score is not associated with MRI traits

**Figure 5:**
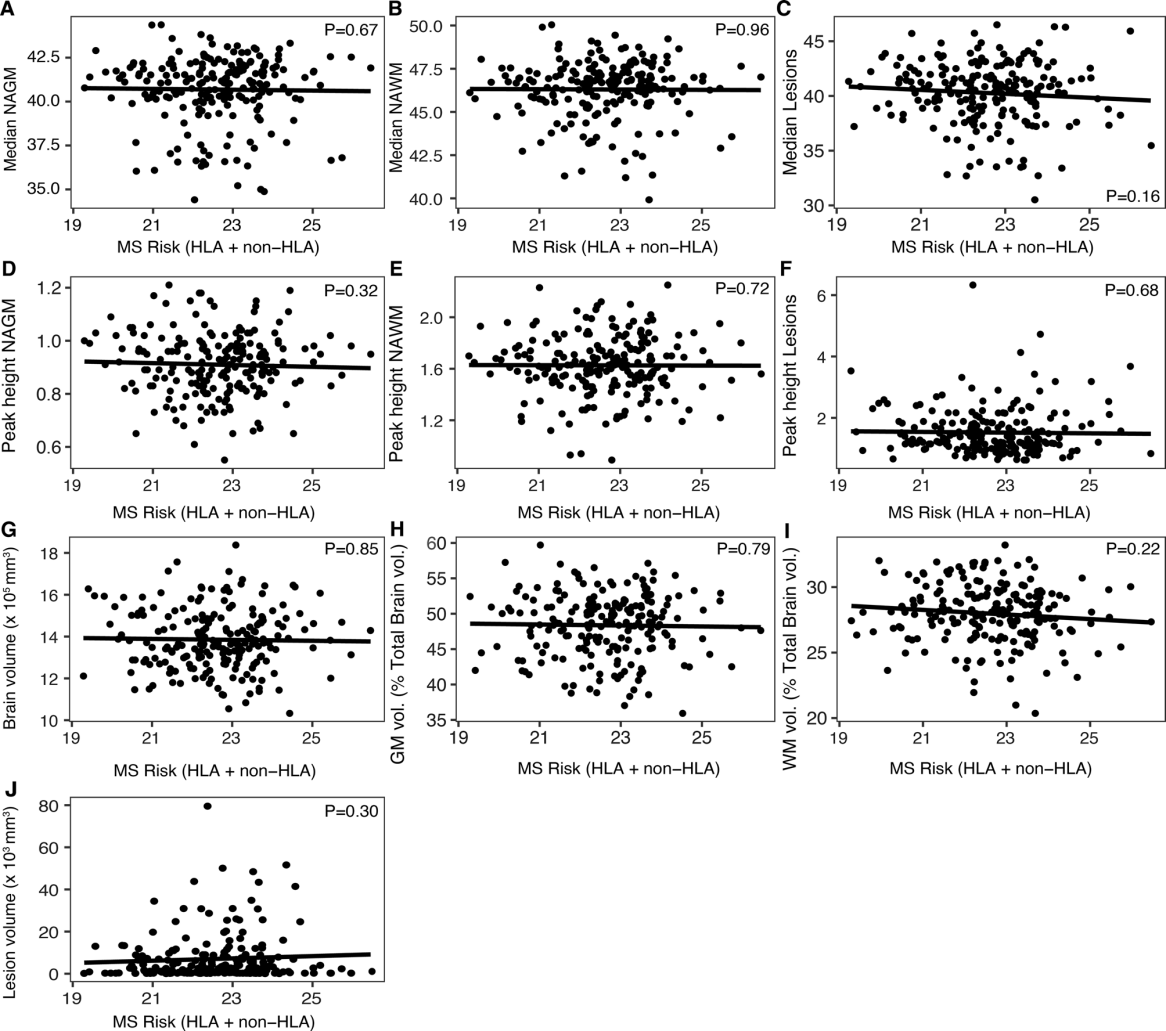
Association of MS genetic risk score with MRI traits. Correlation of (**A**–**C**) median and (**D**–**F**) peak height MTR across tissues as well as (**G**–**J**) volumetric measurements with MS genetic risk score calculated based on known HLA (N = 22) and non-HLA (N = 133) risk variants in the cross-sectional study population (N = 209). P-values were calculated from linear regression including age, gender and protocol as covariates. *NAGM *normal appearing grey matter, *NAWM *normal appearing white matter, *PH *peak height, *GM *grey matter, *WM *white matter, *vol. *volume, *MS *multiple sclerosis, *HLA *human leukocyte antigen (Image generated with Rv3.6.1, https://www.R-project.org).

We subsequently investigated whether, in addition to demographic and clinical variables, genes explaining susceptibility to MS play a role in modulating MTR/volumetric traits. A genetic risk score combining known MS variants was not associated with MTR measures (P ≥ 0.16) or volumetric indices (P ≥ 0.22) (Fig. 5). This conclusion did not change when evaluating separately the non-HLA (P ≥ 0.05) and HLA (P ≥ 0.33) genetic risk score (data not shown). Similarly, the association of individual risk loci with MTR and volumetric measures did not reveal more nominally significant associations than expected by chance [non-HLA: 4.51% (60/1330); HLA 2.7% (6/220)] (Supplementary Table S4–S5).

## Discussion

In this study, we demonstrated the importance of pre-existing inter-patient differences in explaining variation in MTR and volumetric measures among patients. Moreover, there was a moderate correlation of MTR across tissues, especially normal-appearing white and grey matter and to a lesser extent lesions, suggesting a shared underlying pathway. Longitudinally, the generic model including inter-patient (i.e. differences between patients at inclusion) as well as intra-patient (i.e. changes occurring over time in each patient) variation could explain about three quarters of the variance in MTR and volumetric measures. Inter-patient differences were the predominant source of variation and outweighed disease duration or activity reflected by intra-patient variation. Other research approaches could similarly highlight intrinsic heterogeneity in degree of myelination. When lymphocytes from 27 different MS patients were grafted into the demyelinated lesions of nude mice spinal cords, high and low remyelination patterns were observed^22^. A longitudinal follow-up study of the myelin content in lesions of 20 cases with myelin-specific PET tracers documented a large subject-specific heterogeneity in all indices of myelin content change^23^. These findings are consistent with anatomo-pathological observations in post-mortem brain tissue of 51 MS patients. These data showed a profound diversity in remyelination level: a subset displayed extensive remyelination whereas in others remyelination was sparse^9^. Furthermore, many studies have tried to correlate MTR changes over short to medium time intervals with disease evolution^24–27^. However, these observational studies require lengthy patient follow-up as differences between patients and controls are subtle and easily overshadowed by inter-patient differences at baseline^28–30^. The best illustration is a 13-year follow-up cohort of MS patients where only MTR and grey matter fraction at baseline and not the change within the first year of follow-up could predict disability or cognitive decline^27^. Overall, our study does not contradict modest longitudinal change with age and disease duration but demonstrates it is substantially smaller than variation between patients.

The abovementioned literature could only limitedly clarify why patients differ regarding remyelination capacity. The studies mentioned no^22^ or modest associations with age, disease duration^9,23^ and disability level^23^ and the bulk of inter-patient differences remained unexplained. Apart from the association between peak height and age, MTR measures were only limitedly and not consistently associated with clinical or demographical values across tissues in our work. On the other hand, association of age and gender with brain volume measures is well established^31–33^ providing a validation for our dataset. Indeed, together with disease duration, age and gender explained between 30 and 50 percent of the variance in total brain and grey matter volume. Altogether, quantitative MRI parameters might reflect variation between patients in underlying biological processes which could partially be driven by genetics.

Epidemiological studies indeed indicate a genetic basis for variation in MTR, as MTR is decreased to a greater extent in patients with a higher familial burden of disease^34,35^. To date, 200 non-HLA autosomal genetic risk factors for MS, of which 138 are primary, independent effects, as well as 32 statistically independent HLA risk factors have been established^10,36–38^. We summarized the combined effect of these genetic associations in the MS genetic risk score. We previously demonstrated that the MS genetic risk score is correlated with CSF antibody production (HLA burden)^39^. In this study, we did not observe an association of MS genetic risk score with MTR or volumetric measures. This is in line with the absence of genetic associations with disease course (relapsing–remitting versus primary progressive) or severity^40,41^. Similarly, individual HLA alleles did not influence the examined MRI traits. This contrasts with the prominent role HLA-DRB*1501 fulfills in susceptibility. In literature, the association with HLA is debated. Some smaller studies highlight the role of HLA-DRB1*15 on the accumulation of microstructural brain damage^42,43^ whereas others refute this^44,45^. The MS genetic risk factors involve mainly the innate and adaptive immune system^10^, but other pathways may control presentation of disease after onset.

Demyelination has been proposed to be the major pathologic substrate for decreased MTR, but additional pathologic features may contribute to the MTR signal. Increased numbers of enlarged microglia/macrophages contribute to MTR abnormalities in NAWM and even more prominently in lesions^46^. In MS active lesions, these innate immune cells increase already in initial lesion stages and reach their peak in early/late active lesion areas^47^. Microglia-related CSF biomarkers at diagnosis correlate with MTR measured more than 3 years later in a subset of the current study population^13^. In particular, chitotriosidase or CHIT1 levels increase with increasing MTR abnormalities (decreasing MTR) in lesions, and explain 12% of variance in median lesion MTR across patients. Clinical or demographical covariates did not add to the variance explained, and a trend similar to MTR in lesions was seen for MTR in NAGM and NAWM. This is in line with the conclusions of our current work indicating a partially shared biological pathway underlying variation in MTR that is correlated across tissues and inherent to a patient but is not influenced by demographic or clinical variables.

The real-life setting of the study allowed us to collect a large patient population but inherently introduced technical variation since different MRI scanners and MRI scanning protocols were used in daily practice. To take into account this inter-protocol variability, we introduced the five distinct MRI protocols as a covariate in the linear regression model, and only assessed patients that were scanned twice using the same protocol for the longitudinal analysis. Replication of known effects provides validation for this strategy. Furthermore, we acknowledge that next to MT imaging also other informative myelin imaging techniques exist such as myelin water imaging and diffusion tensor imaging^48^. However, MT imaging is technically and logistically the most standardized and feasible technique^48^. Therefore, it is the only technique that allows to be integrated in clinical MRI scanning protocols with typically short time slots.

Our study addresses the determinants of variation in MTR and volumetric traits. We posit that (semi-)quantitative MRI traits change with ongoing disease activity or duration but that this change is modest in comparison to the pre-existing inter-patient differences. Hence, MTR and volumetric indices reflect individual variation in biological processes which is not driven by the known MS genetic susceptibility variants. Additional large-scale genetic studies are required to unravel the pathways associated with MRI heterogeneity.

## Supplementary Information

Supplementary Information 1.

Supplementary Information 2.

Supplementary Information 3.
