# Supplementary material for: Quantitative MRI phenotypes capture biological heterogeneity in multiple sclerosis patients
Source: Sci Rep. 2021 Jan 15;11:1573. doi: 10.1038/s41598-021-81035-8 (PMC7811013; doi:10.1038/s41598-021-81035-8)
Supplement: Supplementary file 1 — Supplementary Information 1. [file 41598_2021_81035_MOESM1_ESM.docx]

Supplementary Information

**Quantitative MRI phenotypes capture biological heterogeneity in multiple sclerosis patients**

Ide Smets, MD, PhD^1,2,3*^, An Goris, MSc, PhD^1,2*^, Marijne Vandebergh, MSc^1,2^, Jelle Demeestere, MD^3^, Stefan Sunaert, MD, PhD^2,4^, Patrick Dupont, MSc, PhD^5^, Bénédicte Dubois, MD, PhD^1,2,3^

^*^These authors contributed equally to this work.

^1^KU Leuven, Department of Neurosciences, Laboratory for Neuroimmunology, 3000 Leuven, Belgium

^2^Leuven Brain Institute KU Leuven, Leuven, Belgium

^3^University Hospitals Leuven, Department of Neurology, 3000 Leuven, Belgium

^4^KU Leuven, Department of Imaging and Pathology, Translational MRI, 3000 Leuven, Belgium

^5^KU Leuven, Department of Neurosciences, Laboratory for Cognitive Neurology, 3000 Leuven, Belgium

Table S1. MRI scanning protocols for 213 patients in the cross-sectional study population.

Patients included in the longitudinal cohort (N = 33) were scanned at least twice using the same protocol D.

| **Protocol** | **Number of patients (%)** | **Scanner** | **Sequence** | **Flip Angle**  **(°)** | **Echo Time (ms)** | **Repetition Time**  **(ms)** | **Pixel Spacing**  **(mm** x **mm)** | **Slice Thickness (mm)** |
| --- | --- | --- | --- | --- | --- | --- | --- | --- |
| **A** | 26  (12.2%) | Achieva | MTR | 15 | 7.99 or 8.0 | 68.62 | 1.00x1.00 | 3 |
|  |  |  | 3D-TFE | 8 | 4.6 | 9.55-9.63 | 0.87x0.87 or 0.98x0.98 | 1.2 |
|  |  |  | 3D-FLAIR | 90 | 339.29-406.27 | 4800 | 1.04x1.04 | 1.12 |
| **B** | 11  (5.2%) | Achieva  dstream | MTR | 10 | 4.59 | 67.81 | 1.00x1.00 | 3 |
|  |  |  | 3D-TFE | 8 | 4.6 | 9.53-9.61 | 0.98x0.98 | 1.2 |
|  |  |  | 3D-FLAIR | 90 | 331.86  - 366.83 | 4800 | 1.04x1.04 | 1.12 |
| **C** | 47  (22.0%) | Ingenia | MTR | 10 | 4.59 or 4.6 | 70.66 | 1.14x1.14 | 3 |
|  |  |  | 3D-TFE | 8 | 4.6 or 4.61 | 9.59-9.77 | 0.98x0.98 | 1.2 |
|  |  |  | 3D-FLAIR | 90 | 343.69-404.31 | 4800 | 1.04x1.04 | 1.12 or 1.2 |
| **D** | 95  (44.6%) | Ingenia | MTR | 10 | 4.59 or 4.6 | 67.75 | 1.00x1.00 | 3 |
|  |  |  | 3D-TFE | 8 | 4.6 or 4.61 | 9.57-9.8 | 0.98x0.98, 0.87x0.87  or 0.78x0.78 | 1.2 |
|  |  |  | 3D-FLAIR | 90 | 323.75-407.83 | 4800 | 1.04x1.04 | 1.12 |
| **E** | 8  (3.8%) | Ingenia | MTR | 10 | 4.59 | 70.66 | 1.14x1.14 | 3 |
|  |  |  | 3D-TFE | 8 | 4.6 or 4.61 | 9.63-9.74 | 0.98x0.98 | 1.2 |
|  |  |  | 3D-FLAIR | 90 | 345.38-392.99 | 4800 | 1.04x1.04 | 1.12 or 1.2 |
| **F** | 26  (12.2%) | Intera | MTR | 10 | 4.59 | 82.58 | 1.00x1.00 | 3 |
|  |  |  | 3D-TFE | 8 | 4.6 | 9.58-9.65 | 0.98x0.98 | 1.2 |
|  |  |  | 3D-FLAIR | 90 | 344.47-392.6 | 4800 | 1.04x1.04 | 1.2 |
| **TOTAL** | 213  (100%) |  |  |  |  |  |  |  |

Table S2. Correlation between MTR parameters.

For MTR, we evaluated the histogram measures median, mean of the middle 90 percentiles of the data (mean90), peak location and peak height in NAWM, NAGM as well as lesions. In all examined tissue classes (**A.** NAGM, **B.** NAWM, **C.** Lesions), there was a high correlation between median and both peak location (r ≥ 0.85) and mean90 (r ≥ 0.99). A moderate correlation between median and peak height MTR (r ≤ 0.57) was also present but significantly less pronounced than the abovementioned correlation. (NAGM = normal appearing grey matter, NAWM = normal appearing white matter)

| NAGM | Median | Mean 90 | Peak height | Peak location |
| --- | --- | --- | --- | --- |
| Median | 1.00 | 0.99 | 0.57 | 0.95 |
| Mean90 |  | 1.00 | 0.61 | 0.93 |
| Peak height |  |  | 1.00 | 0.38 |
| Peak location |  |  |  | 1.00 |

**A.**

**B.**

| NAWM | Median | Mean90 | Peak height | Peak location |
| --- | --- | --- | --- | --- |
| Median | 1.00 | 1.00 | 0.41 | 0.98 |
| Mean90 |  | 1.00 | 0.41 | 0.97 |
| Peak height |  |  | 1.00 | 0.40 |
| Peak location |  |  |  | 1.00 |

**C.**

| Lesions | Median | Mean90 | Peak height | Peak location |
| --- | --- | --- | --- | --- |
| Median | 1.00 | 0.99 | 0.45 | 0.85 |
| Mean90 |  | 1.00 | 0.51 | 0.78 |
| Peak height |  |  | 1.00 | 0.18 |
| Peak location |  |  |  | 1.00 |
| Mean90 |  |  |  |  |

Table S3. Correlations of MRI traits with demographical and clinical parameters.

P-values and effect sizes (i.e. BETA) result from a linear regression analysis with demographic (gender and age) and clinical (disease duration, OCB status, IgG index, MSSS and treatment status) variables. MRI protocol is included as covariates in all analyses and age and gender in analyses with clinical parameters. The nominally significant associations are underlined and associations surviving multiple testing (corrected P ≤ 0.05 corresponds to P ≤ 0.00047) are indicated in bold. (PH = peak height, M = Male, OCB = oligoclonal bands, Neg = negative, IgG = immunoglobulin G, MSSS = Multiple sclerosis Severity Score)

|  | Gender (ref = M) | | Age | | Disease duration | | OCB (ref = Neg) | | Log(IgG) | | MSSS | | Treated (ref = None) | |
| --- | --- | --- | --- | --- | --- | --- | --- | --- | --- | --- | --- | --- | --- | --- |
|  | P | BETA | P | BETA | P | BETA | P | BETA | P | BETA | P | BETA | P | BETA |
| Median MTR NAWM | 0.31 | 0.17 | 0.27 | -0.01 | 0.14 | -0.02 | 0.54 | 0.14 | 0.13 | -0.28 | 0.11 | -0.06 | 0.52 | 0.10 |
| Median MTR NAGM | 0.03 | 0.37 | 0.10 | -0.01 | 0.09 | -0.02 | 0.38 | -0.20 | 0.94 | -0.01 | 0.02 | -0.09 | 0.31 | -0.16 |
| Median MTR Lesions | 0.01 | 0.86 | 0.70 | 0.01 | 0.52 | 0.02 | 0.78 | 0.14 | 0.15 | -0.59 | 0.01 | -0.20 | 0.69 | -0.13 |
| PH MTR NAWM | 0.13 | 0.05 | 2.37E-03 | -4.11E-03 | 0.23 | -2.80E-03 | 0.50 | -0.03 | 0.14 | 0.05 | 0.02 | -0.02 | 0.17 | -0.04 |
| PH MTR NAGM | 2.54E-03 | 0.05 | **2.98E-04** | -2.37E-03 | 0.14 | -1.64E-03 | 0.07 | -0.04 | 0.34 | 0.02 | 0.01 | -0.01 | 4.87E-04 | -0.05 |
| PH MTR Lesions | 0.03 | 0.24 | 4.87E-03 | -0.01 | 0.30 | -0.01 | 0.75 | -0.05 | 0.65 | -0.06 | 0.01 | -0.07 | 0.01 | -0.28 |
| Brain volume(mm^3^) | **1.67E-21** | -193072.04 | 0.38 | 649.07 | 0.14 | -1878.43 | 0.30 | 26089.06 | 0.82 | -4695.19 | 0.92 | -422.05 | 0.88 | 2609.68 |
| WM volume (%) | 0.32 | 0.33 | 0.53 | -0.01 | 0.05 | -0.05 | 0.13 | -0.69 | 0.92 | -0.03 | 0.21 | -0.10 | 0.07 | -0.58 |
| GM volume (%) | 3.58E-04 | 1.99 | **4.57E-16** | -0.20 | 0.76 | -0.01 | 0.82 | 0.18 | 0.91 | 0.07 | 0.11 | -0.21 | 0.22 | -0.65 |
| Lesion volume (mm^3^) | 0.27 | -1860.49 | 0.06 | 125.58 | 0.02 | 265.81 | 0.70 | 899.62 | 0.24 | 2233.07 | **1.19E-04** | 1478.56 | 0.50 | 1086.97 |

Table S4. Association results from the univariate analysis for the 133 known non-HLA MS genetic risk factors.

P-values result from the SNPTEST analysis and are depicted in column 5 until 14 for each of the 10 examined MRI traits. Nominally significant associations are underlined. (MTR = magnetization transfer ratio, SNP = single nucleotide polymorphism, NAWM = normal appearing white matter, NAGM = normal appearing grey matter, WM = white matter, GM = grey matter)

SupplS4.xlsx

Table S5. Association results from univariate analysis for the 22 known HLA MS risk factors.

P-values result from the SNPTEST analysis and are depicted in column 2 until 11 for each of the 10 examined MRI traits. Nominally significant associations are underlined. (MTR = magnetization transfer ratio, NAWM = normal appearing white matter, NAGM = normal appearing grey matter, PH = peak height, WM = white matter, GM = grey matter, HLA = human leukocyte antigen)

SupplS5.xlsx
